# Prospective SPECT-CT Organ Dosimetry-Driven Radiation-Absorbed Dose Escalation Using the In-111 (^111^In)/Yttrium 90 (^90^Y) Ibritumomab Tiuxetan (Zevalin^®^) Theranostic Pair in Patients with Lymphoma at Myeloablative Dose Levels

**DOI:** 10.3390/cancers13112828

**Published:** 2021-06-06

**Authors:** Richard L. Wahl, Eric C. Frey, Heather A. Jacene, Brad S. Kahl, Steven Piantadosi, Jesus A. Bianco, Richard J. Hammes, Miah Jung, Wayne Kasecamp, Bin He, George Sgouros, Ian W. Flinn, Lode J. Swinnen

**Affiliations:** 1 Sidney Kimmel Comprehensive Cancer Center, Johns Hopkins University, Baltimore, MD 21287, USA; efrey@jhmi.edu (E.C.F.); spiantadosi@bwh.harvard.edu (S.P.); miah.jung@fda.hhs.gov (M.J.); bhe@jhmi.edu (B.H.); iflinn@tnonc.com (I.W.F.); lswinne1@jhmi.edu (L.J.S.); 2 Division of Nuclear Medicine, Russell H. Morgan Department of Radiology and Radiological Sciences, Johns Hopkins University, Baltimore, MD 21205, USA; hjacene@bwh.harvard.edu (H.A.J.); jbianco@uwisc.edu (J.A.B.); wkaseca@comcast.net (W.K.); gsgouros@jhmi.edu (G.S.); 3 Mallinckrodt Institute of Radiology, Washington University School of Medicine, St. Louis, MO 63130, USA; 4 Radiopharmaceutical Imaging and Dosimetry, LLC, Baltimore, MD 21231, USA; 5 Dana-Farber Cancer Institute, Harvard Medical School, Boston, MA 02115, USA; 6 Departments of Medicine and Radiology, University of Wisconsin Medical School, Madison, WI 53706, USA; bkahl@wustl.edu (B.S.K.); rjhammes@gmail.com (R.J.H.); 7 Division of Medical Oncology, Department of Internal Medicine, Washington University School of Medicine, St. Louis, MO 63130, USA; 8 Department of Surgery, Brigham and Women’s Hospital, Harvard Medical School, Boston, MA 02115, USA; 9 Sarah Cannon Research Institute, Nashville, TN 37203, USA

**Keywords:** radioimmunotherapy, dosimetry, quantitative SPECT imaging, anti CD20, lymphoma, theranostics

## Abstract

**Simple Summary:**

We prospectively evaluated the feasibility of SPECT-CT/planar organ dosimetry-based radiation dose escalation radioimmunotherapy in patients with recurrent non-Hodgkin’s lymphoma using the theranostic pair of ^111^In and ^90^Y anti-CD20 ibritumomab tiuxetan (Zevalin^®^) at myeloablative radiation-absorbed doses with autologous stem cell support. Unlike most routine dose escalation approaches, our approach used patient-individualized measurements of organ radiation absorbed dose from the tracer study, with patient-specific adjustments of the injected therapy dose to deliver a pre-specified radiation absorbed dose to the liver. Our approach was feasible, stem cell engraftment was swift, resulted in an 89% tumor response rate in treated patients, demonstrated over 3 fold variability in liver dosimetry/injected activity among patients, allowed us to exceed the FDA approved administered activity by over 5 fold and demonstrated the normal liver maximum tolerated dose to exceed 28 Gy. Dose escalation was not continued due to lack of drug availability. With modern dosimetry approaches, patient specific dosimetry-driven radiation dose escalation is feasible, allows adjustment of administered activity for heterogeneous pharmacokinetics and allows marked dose escalation vs. non-dosimetry driven approaches.

**Abstract:**

Purpose: We prospectively evaluated the feasibility of SPECT-CT/planar organ dosimetry-based radiation dose escalation radioimmunotherapy in patients with recurrent non-Hodgkin’s lymphoma using the theranostic pair of ^111^In and ^90^Y anti-CD20 ibritumomab tiuxetan (Zevalin^®^) at myeloablative radiation-absorbed doses with autologous stem cell support. We also assessed acute non-hematopoietic toxicity and early tumor response in this two-center outpatient study. Methods: 24 patients with CD20-positive relapsed or refractory rituximab-sensitive, low-grade, mantle cell, or diffuse large-cell NHL, with normal organ function, platelet counts > 75,000/mm^3^, and <35% tumor involvement in the marrow were treated with Rituximab (375 mg/m^2^) weekly for 4 consecutive weeks, then one dose of cyclophosphamide 2.5 g/m^2^ with filgrastim 10 mcg/kg/day until stem cell collection. Of these, 18 patients with successful stem cell collection (at least 2 × 10^6^ CD34 cells/kg) proceeded to RIT. A dosimetric administration of ^111^In ibritumomab tiuxetan (185 MBq) followed by five sequential quantitative planar and one SPECT/CT scan was used to determine predicted organ radiation-absorbed dose. Two weeks later, ^90^Y ibritumomab tiuxetan was administered in an outpatient setting at a cohort- and patient-specific predicted organ radiation-absorbed dose guided by a Continuous Response Assessment (CRM) methodology with the following cohorts for dose escalation: 14.8 MBq/kg, and targeted 18, 24, 28, and 30.5 Gy to the liver. Autologous stem cell infusion occurred when the estimated marrow radiation-absorbed dose rate was predicted to be <1 cGy/h. Feasibility, short-term toxicities, and tumor response were assessed. Results: Patient-specific hybrid SPECT/CT + planar organ dosimetry was feasible in all 18 cases and used to determine the patient-specific therapeutic dose and guide dose escalation (26.8 ± 7.3 MBq/kg (mean), 26.3 MBq/kg (median) of ^90^Y (range: 12.1–41.4 MBq/kg)) of ibritumomab tiuxetan that was required to deliver 10 Gy to the liver. Infused stem cells engrafted rapidly. The most common treatment-related toxicities were hematological and were reversible following stem cell infusion. No significant hepatotoxicity was seen. One patient died from probable treatment-related causes—pneumonia at day 27 post-transplant. One patient at dose level 18 Gy developed myelodysplastic syndrome (MDS), 4 patients required admission post-^90^Y RIT for febrile neutropenia, 16/18 patients receiving ^90^Y ibritumomab tiuxetan (89%) responded to the therapy, with 13 CR (72%) and 3/18 PR (17%), at 60 days post-treatment. Two patients had progressive disease at sixty days. One patient was lost to follow-up. Median time to progression was estimated to be at least 13 months. MTD to the liver is greater than 28 Gy, but the MTD was not reached as the study was terminated due to unexpected discontinuation of availability of the therapeutic agent. Conclusions: Patient-specific outpatient ^90^Y ibritumomab tiuxetan RIT with myeloablative doses of RIT up to a targeted 30.5 Gy to the liver is feasible, guided by prospective SPECT/CT + planar imaging with the theranostic pair of ^111^In and ^90^Y anti-CD20, with outpatient autologous stem cell transplant support. Administered activity over 5 times the standard FDA-approved activity was well-tolerated. The non-hematopoietic MTD in this study exceeds 28 Gy to the liver. Initial tumor responses were common at all dose levels. This study supports the feasibility of organ dosimetry-driven patient-specific dose escalation in the treatment of NHL with stem cell transplant and provides additional information on the radiation tolerance of the normal liver to radiopharmaceutical therapy.

## 1. Introduction

Anti-CD20-targeted monoclonal antibodies, both unlabeled and radiolabeled, are active agents in the treatment of B-cell lymphomas [1,2]. The addition of radioactivity to the anti-CD20 antibody increases therapeutic efficacy over the unlabeled antibody [3,4]. The FDA has approved two anti-CD20 radio-immunotherapies for follicular lymphomas: ^131^I tositumomab plus unlabeled tositumomab (Bexxar^®^, Philadelphia, PA, USA) and rituximab plus ^90^Y ibritumomab tiuxetan (Zevalin^®^, East Windsor, NJ, USA). Their administered radioactivity doses, and quite likely their efficacy, are limited by hematopoietic toxicity, predominantly thrombocytopenia [5]. ^131^I rituximab has similar efficacy and dose, limiting hematopoietic toxicities [6]. While anti-CD20 radio-immunotherapies are effective and safe, their utilization at non-myeloablative doses has been relatively limited given the availability of other therapeutic approaches and other factors, with only 44 treatments reported for Zevalin in a European therapy survey in 2015 [7,8]. Indeed, Bexxar is no longer commercially available, mainly due to low commercial sales.

Lymphomas, particularly follicular lymphomas, are not generally cured with conventional therapies. Consolidative ^131^I Bexxar radioimmunotherapy after conventional chemotherapy has recently shown very promising outcomes [9]. Thus, there is renewed interest in the use of radioimmunotherapy and other approaches to improve outcomes of patients with lymphoma.

Using modern SPECT/CT dosimetry approaches, baseline ^131^I tositumomab dosimetry scans predict radiation dosimetry estimates on post-therapy scans. Dosimetry also showed increased radiation-absorbed dose (>200 cGy) to lymphomas to be significantly associated with longer progression-free survival [10]. Thus, there is strong evidence to suggest that increasing tumor radiation-absorbed dose would enhance tumor response.

To substantially increase radiation-absorbed doses to tumors requires dose escalation with stem cell transplant support to mitigate hematopoietic toxicities. ^131^I tositumomab has been used at myeloablative doses guided by planar dosimetry with stem cell transplants, with encouraging results [11,12,13].

The radiation dosimetry profiles differ between ^131^I tositumomab and ^90^Y ibritumomab tiuxetan, with a substantially higher radiation-absorbed dose delivered to the liver by ^90^Y ibritumomab tiuxetan relative to ^131^I tositumomab. This is likely because radiometals such as indium and yttrium are retained in the liver to a greater extent than radioiodine [14]. Wiseman et al. reported a median estimated radiation-absorbed dose of 3.4 Gy to liver (range 1.2–7.8 Gy), 2.6 Gy to lungs (range 0.72–4.4 Gy), and 0.38 Gy to kidneys (range 0.07–0.61 Gy) for the standard dosing regimen [15]. Considerable patient-to-patient variability in dosimetry to normal tissues has been seen with Zevalin radioimmunotherapy [15,16,17].

Given that the lungs, or much less commonly the kidneys, received the highest radiation-absorbed dose with ^131^I anti-CD20 therapy, while the liver received the highest estimated radiation-absorbed dose in ^90^Y ibritumomab therapies, the non-hematopoietic toxicity of higher-dose ^90^Y ibritumomab tiuxetan therapy with stem cell support might be expected to be different than that of high-dose ^131^I tositumomab. In addition, the pure high-energy beta emission of ^90^Y delivers the vast majority of its energy within the patient without a gamma emission, a practical advantage vs. ^131^I, which has a 364 keV gamma emission, necessitating substantial radiation safety precautions, including inpatient stays. Further, there is relatively little ^90^Y excretion from the patient when ^90^Y monoclonal antibodies are administered, in contrast to the substantial urinary excretion with ^131^I. Thus, the radiation safety profile for higher-dose ^90^Y myeloablative RIT with autologous stem cell transplant support lends itself to outpatient therapy. The higher beta energy of ^90^Y vs. ^131^I is expected to deliver radiation more uniformly to large tumor foci, also a potential advantage for RIT with ^90^Y.

Two groups have reported on the use of myeloablative doses of Y-90 Ibritumomomab Tiuxetan in the treatment of NHL. In both studies, dose escalations were based on escalating mBq/kg dosing cohorts informed by planar dosimetry imaging methods, not including SPECT/CT. Target liver radiation-absorbed doses were not to exceed 20 Gy [16]. Planar imaging is not as accurate as SPECT/CT imaging, indeed there is considerable variability among dosimetry approaches on the same datasets [16].

It is increasingly possible to perform accurate quantitative SPECT dosimetry with CT-based attenuation correction. One approach is to perform multiple SPECT/CT studies over time [10]. Another approach is to ascertain the shape of the time-activity curve for an organ from planar imaging and adjust its precise height using SPECT/CT performed at one or more time points [18,19]. Prospectively performing organ dosimetry in individual patients has technical and logistical challenges. In the present study, we sought to determine the feasibility of such a real-time approach in patients with lymphoma to allow for substantial prospective precision dose escalation.

Thus, we conducted a phase I dose escalation study of ^90^Y ibritumomab tiuxetan using ^111^In ibritumomab tiuxetan as a surrogate for ^90^Y ibritumomab tiuxetan, for treatment planning in patients with CD20-positive NHL, guided by more accurate organ-specific dosimetry determined by hybrid SPECT/CT and planar imaging. The goals of our study were to determine (a) the feasibility of SPECT/CT + planar prospective real-time dosimetry for guiding individual patient administered radioactivity levels, (b) the feasibility of fully outpatient RIT with ASCT support, (c) to estimate the non-hematological MTD of this treatment approach, especially as related to radiation-induced hepatotoxicity (up to 30.5 Gy) and marrow re-engraftment, and (d) to preliminarily assess the therapeutic activity of this treatment regimen in patients with resistant or refractory CD20-positive, non-Hodgkin’s lymphoma.

## 2. Methods

The study was performed as a prospective, two-center, dose-escalation study of ^90^Y ibritumomab tiuxetan, with dose escalation based on prospective patient-specific organ dosimetry determined from ^111^In ibritumomab tiuxetan imaging. The primary operational goal of the study was to determine if precision, prospective, SPECT-CT organ dosimetry-based outpatient myeloablative radioimmunotherapy, with autologous stem cell transplantation (ASCT), in a two-center study was logistically feasible. The primary clinical goal was to determine the non-hematopoietic MTD of 90Y ibritumomab tiuxetan, with rituximab in vivo purging and ASCT support, using the precision dosing method. The study was conducted according to the guidelines of the Declaration of Helsinki, was approved by the IRBs of the two participating sites (IRB# NA_00036237), and written informed consent was secured for all patients. The trial was initiated before the clinicaltrials.gov (accessed on 5 June 2021) registration procedure was required.

Inclusion criteria: Eligible patients had relapsed or refractory CD20-positive low-grade, mantle cell, or diffuse large-cell NHL, rituximab sensitivity (rituximab-sensitive defined as at least partial response to rituximab or rituximab-containing regimen and response duration of at least 6 months), and age > 18 years. In addition, patients had to have acceptable function of bone marrow (platelet count ≥ 75,000/mm^3^, WBC ≥ 3000/mm^3^, lymphomatous marrow involvement < 35%), and adequate liver, heart, and kidney function (creatinine ≤ 2 mg/dL, direct bilirubin ≤ 2 mg/dL, LVEF ≥ 45%, DLCO > 50%). Patients had to have an anatomically measurable tumor.

Theranostic regimen: In the first phase of the theranostic regimen, rituximab (375 mg/m^2^) was administered weekly for 4 consecutive weeks, followed by a single dose of cyclophosphamide (2.5 g/m^2^). Filgrastim 10 mcg/kg was administered after cyclophosphamide until stem cell collection. Patients with successful stem cell collection (at least 2 × 10^6^ CD34 cells/kg) proceeded to RIT. In the second phase of the treatment, on Day 1, patients were given a dosimetric dose of ^111^In ibritumomab tiuxetan (185 MBq), followed by planar scans and blood sampling at 0–1, 4, 24, 72, and 144 h post-tracer injection and a quantitative SPECT/CT at 24 h. Hybrid planar-SPECT quantification and MIRD dosimetry with liver mass corrections were used to evaluate organ doses. On Day 15, ^90^Y ibritumomab tiuxetan was administered at a cohort-specific radiation-absorbed dose based on the results of the organ dosimetry. Stem cell infusion occurred when estimated marrow dose rate was <1 cGy/hour. Estimates of bone marrow radiation-absorbed dose were based on the most conservative estimates of dose obtained from blood and image-based marrow dosimetry.

**Figure 1:**
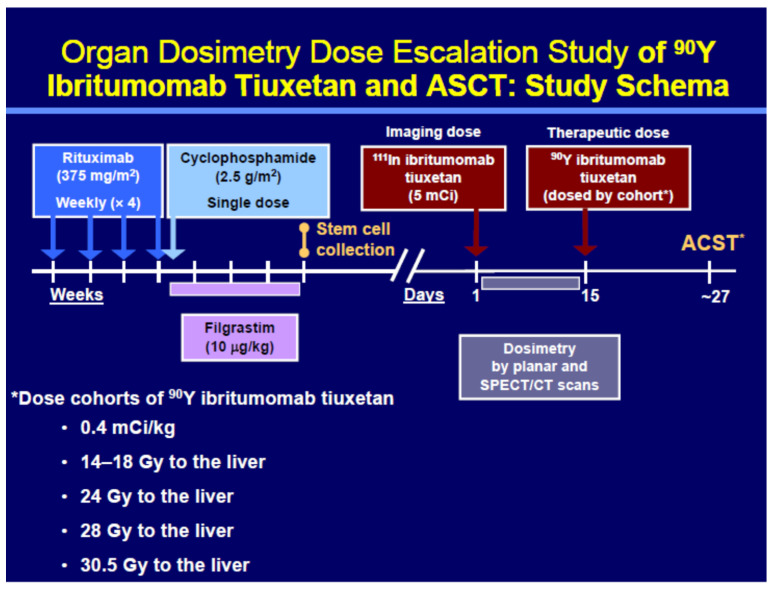
Study schema. Patients had to have adequate stem cell harvests to move forward to the imaging and ^90^Y ibritumomab tiuxetan therapy arm.

The study schema is shown in Figure 1. The theranostic procedures were performed at two different physical sites, though dosimetry was calculated at a single site, Johns Hopkins University School of Medicine. A total of 13 patients were treated with ^90^Y ibritumomab tiuxetan at Johns Hopkins University, Baltimore, MD, and 5 at the University of Wisconsin, Madison, WI, USA.

A modified continual reassessment method (CRM) dose escalation was used to determine the target radiation-absorbed dose for each cohort. The CRM was initiated by assuming a 10% response would occur from 10 Gy and a 90% response would occur from 35 Gy delivered to the liver. The probability of toxicity was assumed to follow a logistic dose response model which was updated after each cohort. The initial cohort received the standard 14.8 MBq/kg (0.4 mCi/kg) dose of ^90^Y, irrespective of predicted organ dose. Dose cohorts determined by this method were 14.8 MBq/kg and (maximum liver-absorbed dose of ^90^Y RIT) 14–18, 24, 28, and 30.5 Gy in Cohorts 1–5, respectively [20].

### 2.1. Patient Population

The patient population is shown in Table 1. Median patient age was 55 and median number of prior therapies was 3. Follicular lymphoma, transformed follicular lymphoma, and mantle cell lymphomas were the most common histologies, and 24 patients were entered in the study. Six patients failed to mobilize adequate numbers of stem cells and did not receive ^111^In dosimetric/biodistribution scans nor ^90^Y RIT.

### 2.2. Imaging and Dosimetry

The predicted organ doses and the patient-specific administered radioactivity levels for each patient were calculated using a combination of planar whole-body images acquired at five time points and two SPECT/CT images acquired at one time point. The imaging was performed with a GE Millenium VG/Hawkeye SPECT/CT system with a 1.5875 cm thick crystal at both performance sites. The whole-body images were acquired at, nominally, 0–2, 4, 24, 72, and 144 h post-infusion of ^111^In ibritumomab tiuxetan. Images were acquired in 14% wide-energy windows centered on both ^111^In photo-peaks and corrected for scatter using the TEW method with a 152 keV ± 4% scatter window below the lower photo-peak and a 205 keV ±10% scatter window between the two photo-peaks. A source with known activity was included in the whole-body scan and used to adjust for sensitivity variations, and planar images of the source were used to measure the system’s sensitivity for SPECT image quantification. Whole-body images were scatter-corrected using the TEW method (assuming no counts above the upper photo-peak), the geometric mean from opposing views was calculated, and the resulting voxel values converted to activity using the counts from the first time point and the assumption that all activity was retained in the body at the first time point. Details and evaluation of the quantitative methods are provided in several references [18,19,21,22]. SPECT/CT of the thorax and upper abdomen was performed at 24 h post-injection and reconstructed using vendor supplied software that included attenuation and TEW scatter corrections. These regions of the body were selected to allow estimation of the activity distribution in the dosimetrically important organs, i.e., the lungs and liver.

The predicted radiation-absorbed dose and the administered therapeutic dose for each patient were calculated using the above images using hybrid SPECT/planar methodology [19,22]. Whole-organ volumes of interest (VOIs) were manually defined using both the CT and SPECT images and quantified activity in the major organs. It was sometimes difficult to determine which voxels belong to the liver due to the limited resolution of the SPECT/CT images and high uptake at 24 h post-administration in the spleen and heart. In this case, we included only voxels in the liver VOIs that were certain to represent liver tissue. Projections of the SPECT organ VOIs were used to guide manual definition of regions-of-interest in the planar geometric mean images. The whole-body geometric mean images were quantified using the assumption that all the activity was present in the 0–2 h images and corrected for sensitivity variations using a VOI defined over the standard source image. Time-activity curves were generated from the planar images, adjusted for radioactive decay to represent ^90^Y activity, fit with mono- or bi-exponentials, and rescaled so that the activity for each organ was equal to the activity for that organ at the time of the SPECT/CT acquisition. These curves were integrated and divided by the administered ^111^In activity to give the residence time (time-integrated activity coefficients) for each organ. Specific doses (radiation-absorbed dose per unit administered activity) were calculated from the residence times using MIRD methodology, as implemented in the MIRDose 3.0 code. The appropriate gender for each patient was used. The liver dose was mass corrected, as described above, based on the volume of the liver VOI, an assumed density of 1.0 g/cc for the liver, and the standard organ mass for the appropriate gender. This method is equivalent to using a VOI smaller than the full liver to estimate the activity concentration in the liver and estimating the total liver activity from this activity concentration and the liver volume based on the standard liver volume of the MIRD phantom. The accuracy of this methodology has been validated in [23]. The target administered activity needed to give the target dose was estimated by dividing the target liver dose for the cohort by the specific liver activity, calculated as described above. Images, ROIs, VOIs, and dose calculations from the remote performance site were reviewed centrally at Johns Hopkins.

**Figure 2:**
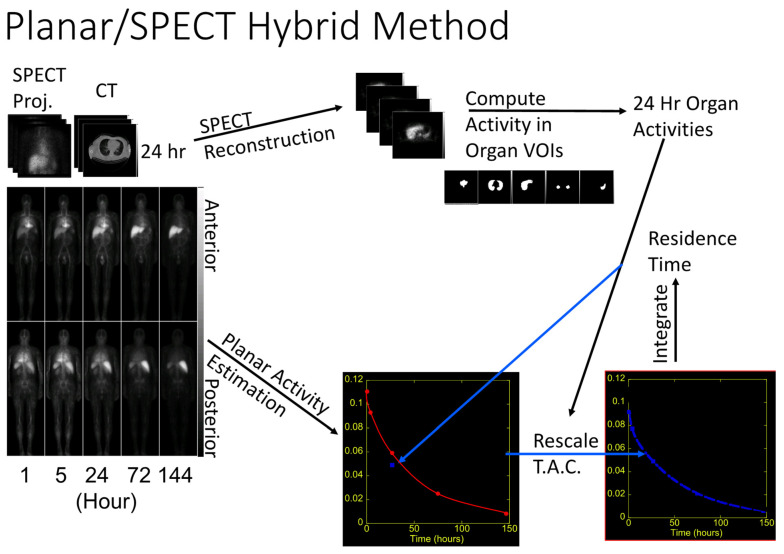
SPECT/CT + planar hybrid dosimetry method. The absolute radioactivity concentration in normal tissues is determined by SPECT/CT imaging performed 24 h after a tracer injection of 5 mCi of ^111^In ibritumomab tiuxetan. The shape of the time-activity curve of radioactivity clearance is defined by sequential whole-body planar conjugate view images. MIRD formalisms are applied with regions-of-interest and organ volumes informed by the CT from the SPECT/CT.

All calculations were completed and reviewed with the PI, and the patient-specific treatment dose was administered at approximately 15 days post-administration of the planning dose. Representative whole-body images and SPECT/CT images with regions of interests selected are shown in Figure 2, which illustrates the theranostic dosimetry schema.

### 2.3. Radiolabeling and Dose Infusion

The ^111^In ibritumomab tiuxetan and ^90^Y ibritumomab tiuxetan were prepared from FDA-approved kits supplied by Biogen/IDEC. The kits were designed to provide a 185 MBq (5 mCi) ^111^In dose for imaging or a maximum of 1.18 GBq (32 mCi)/kit of ^90^Y. To administer therapeutic doses greater than 1.18 GBq, additional kits were required, each supplying 1.18 GBq of ^90^Y activity. A commercial nuclear pharmacy formulated the doses (Cardinal Health, Dublin, OH, USA). Given that the patients had received weekly infusions of rituximab for each of the 4 weeks prior to the dosimetric dose, no unlabeled rituximab was administered immediately prior to infusion of the dosimetric or the therapeutic doses. This is in contrast to the FDA-approved clinical therapy approach, in which an infusion of 250 mg/m^2^ rituximab is given over 1–2 h immediately prior to administration of each of the dosimetric or therapeutic doses. In all cases, patients were treated as outpatients, as their radiation emissions (bremsstrahlung), as assessed by measured radiation levels at one meter following therapy, allowed for them to remain as outpatients in compliance with state and NRC radiation safety guidelines. No post-treatment dosimetric imaging was performed.

### 2.4. Stem Cell Reinfusion

Stem cells were reinfused on an outpatient basis when it was predicted that the radiation-absorbed dose to the stem cells would be less than 1 cGy/h. This prediction was based on the higher predictions made from marrow dosimetry calculations based on blood draws and imaging. The blood-based dosimetry involved sequential measurements of residual blood radioactivity following treatment (obtained on the days of imaging), and the image-based dosimetry used the same methodology as for other organs but with VOIs drawn on lumbar vertebrae and the pelvis. Conservative estimates were used so as to minimize the possibility of radiation damage to the reinfused stem cells. The number of stem cells infused varied based on the success of the stem cell mobilization and collection steps with re-engraftment from 7 to 19 days post-reinfusion.

### 2.5. Follow-Up

The RIT and the stem cell infusions were performed as outpatients; however, patients were closely followed for signs and symptoms of toxicity as they rapidly became lymphopenic and neutropenic following higher doses of ^90^Y anti-CD20. Patients were managed by the marrow transplantation unit, and if neutropenic fevers or other side effects developed that were not readily manageable as outpatients, patients were admitted to the hospital for additional supportive therapy. Patients were followed regularly for hematological and systemic toxicities, with a special emphasis on hepatic toxicity. Imaging assessments, typically whole-body CT, were performed at 60 days post-^90^Y RIT and also at approximately 6-month intervals following treatment with response assessed by the International Working Group criteria (IWG), unless there were clinical signs suggesting earlier progression. The study was terminated early due to sale of the drug provider, meaning that completion of the dose escalation, as well as long-term follow-up data are not available.

## 3. Results

### 3.1. Dosimetry

**Figure 3:**
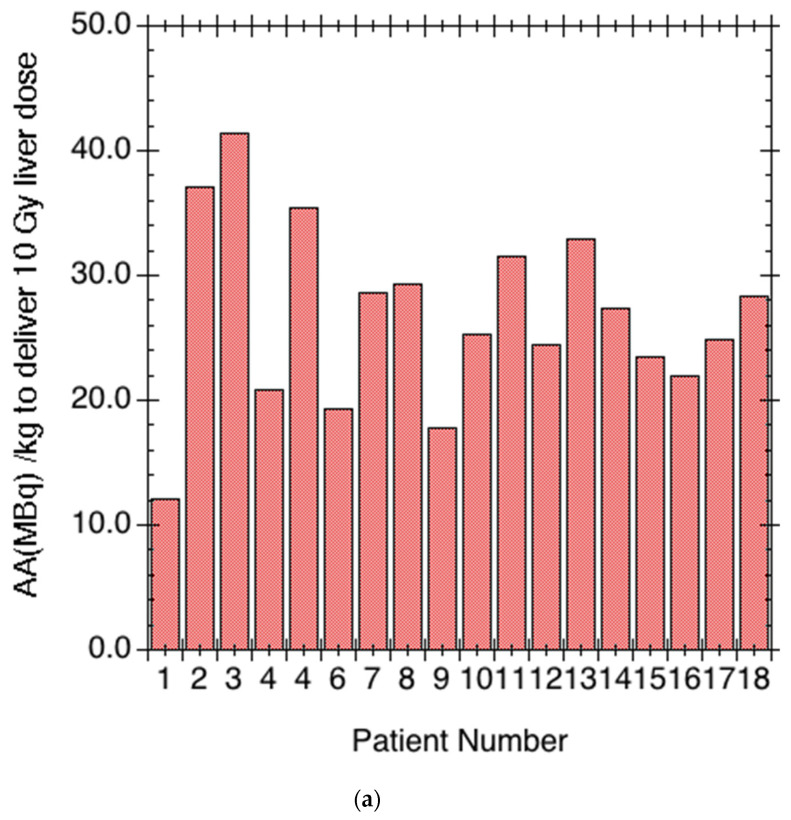

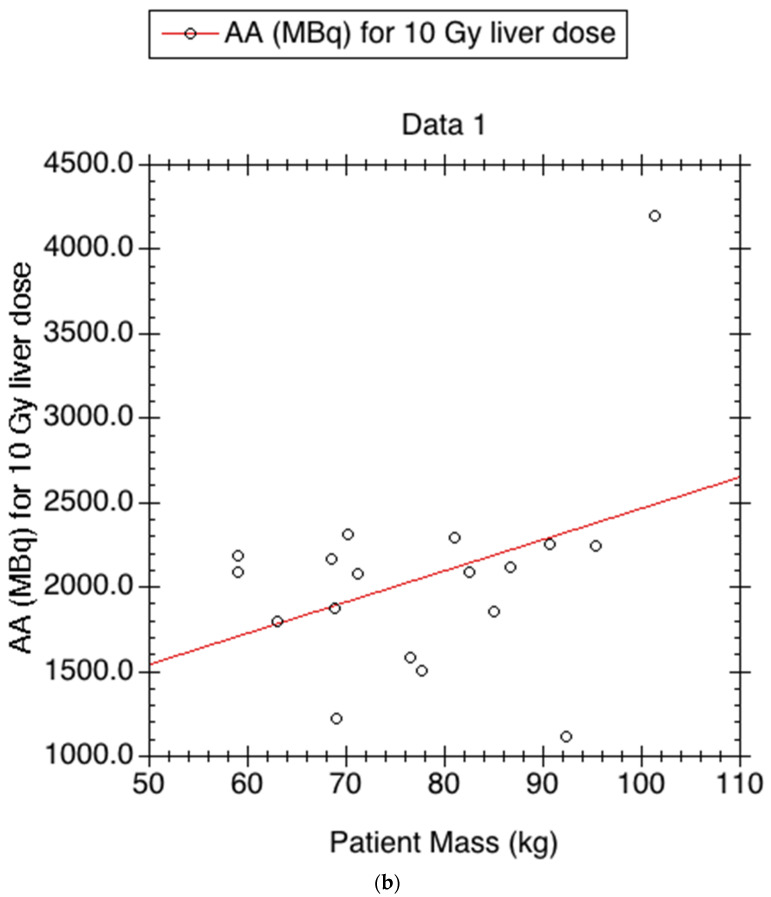
(**a**) Plot of activity per unit mass (MBq/kg) required to deliver 10 Gy to the liver using ^90^Y ibritumomab tiuxetan by patient number. Note that there is an approximately 3.5-fold variability between the highest and lowest required activities of ^90^Y/kg. (**b**) Plot of administered activity to deliver 10 Gy to the liver vs. body mass. Note there is only a modest (r = 0.37) correlation between these values, supporting the need for patient-specific dosimetry beyond adjusting the dose for patient weight.

The prospective planar + SPECT/CT dosimetry methods were successfully performed at a central dosimetry site in all 18 patients who mobilized their stem cells and who were suitable for RIT. In all cases, the liver or spleen were the tissues that were predicted to receive the highest radiation-absorbed dose from the therapy. However, splenic dose was not considered dose-limiting as it was a potential site of tumor involvement. The administered activity per unit mass required to deliver a uniform 10 Gy dose to the liver was 26.8 ± 7.3 MBq/kg of ^90^Y (range: 12.1–41.4 MBq/kg) of ibritumomab tiuxetan, indicating considerable heterogeneity among patients (Table 2, Figure 3a,b). The prospective estimation of predicted therapeutic organ radiation-absorbed dose was optimized for the lungs, liver, kidneys, and the heart. Organs not included in the two SPECT/CT fields-of-view could only be assessed for dosimetry by planar methods, but these organs were not dose-limiting. As the focus of the study was not to estimate tumor radiation-absorbed dose, tumor doses were not calculated. Organ dosimetry estimates and injected activity levels and the target infused activity level are shown in Table 2. All patients were treated as outpatients as radiation emission levels were low enough to comply with local and NRC/state regulations, allowing for outpatient release following therapy.

### 3.2. Marrow Reconstitution

Six patients did not adequately mobilize stem cells and could not be treated with ^90^Y ibritumomab tiuxetan. In the 18 patients treated with ^90^Y, the days to engraftment post-ASCT were, for Cohort 1 (14.8 MBq/kg): 15 days median (range 11–19), Cohort 2 (14–18 Gy): 15.5 days median (range 14–18), Cohort 3 (24 Gy): 10.5 days median (range 10–14), and Cohort 4 (28 Gy): 12 days, range 9–14. The time to engraftment did not appear to depend on the radiation-absorbed dose but seemed somewhat related to the number of infused stem cells.

### 3.3. Toxicities

In the 18 patients who adequately mobilized stem cells, all patients engrafted successfully post-RIT. Notable Phase 1 (mobilization) Grade 3–4 toxicities included: leukopenia, neutropenia, thrombocytopenia, nausea/vomiting, febrile neutropenia, and elevated creatinine. Notable Phase 2 (^111^In Ibritumomab Tiuxetan, ^90^Y ibritumomab tiuxetan, ASCT) Grade 3–4 toxicities included leukopenia, neutropenia, thrombocytopenia, nausea/vomiting, febrile neutropenia, anemia, headache, myalgia, elevated creatinine, elevated AST, elevated ALT, infection with neutropenia, and diarrhea. No significant hepatotoxicity was observed, despite the liver generally receiving a higher absorbed dose of radiation than any other organ. One patient had a rise in serum bilirubin in excess of 3, but this was associated with tumor progression in the liver and porta hepatis. No cardiopulmonary toxicity directly attributable to the therapy was seen, though one patient died on day 27 with pneumonia.

Therapy was delivered in the outpatient setting. Six patients were hospitalized after receiving ^90^Y ibritumomab tiuxetan: four for febrile neutropenia. Admission was also required in one patient for progressive disease and another for diarrhea. One patient died from progressive disease (day 3 post-transplant), a second patient died of complications due to pneumonia (day 27 post-transplant), while another patient died from progressive disease > 12 months post-ASCT, and a fourth patient died from progressive disease but never received Y2B8 or ASCT. One patient at the 18 Gy dose level developed myelodysplastic syndrome (MDS). One patient at 24 Gy developed Grade 2 auto-GVHD (graft vs. host disease) localized to the mouth. Additional long-term follow-up of patients was not available.

### 3.4. Therapeutic Response

Eighteen patients qualified for evaluation at sixty days post-transplant, thirteen patients achieved a CR, three had a PR, while two had PD. The frequency and duration of response is shown in Table 3 by dose cohort. There was not an obvious trend to a higher response rate nor longer PFS apparent across dose ranges (Table 3).

## 4. Discussion

We demonstrated the feasibility of a prospective, precision, SPECT-CT + planar organ dosimetry-based, dose escalation, outpatient myeloablative radioimmunotherapy with autologous stem cell transplantation (ASCT) theranostic regimen in patients with extensively pre-treated non-Hodgkin’s lymphoma. This organ dosimetry method was feasible in the short window of time following infusion of the ^111^In ibritumomab tiuxetan, in a two-center study using a centralized, standardized dosimetry regimen using comparable imaging equipment.

Six of the twenty-four patients entered in the study did not adequately mobilize stem cells sufficiently to advance to transplant, thus eighteen patients received a therapeutic dose of ^90^Y ibritumomab tiuxetan. The pure beta energy emission of ^90^Y and the limited excretion of ^90^Y from the body (approximately 5% urinary excretion) allowed this theranostic approach to be performed within the guidelines of applicable local radiation safety rules without hospitalization for radiation safety considerations [15]. Four of the eighteen patients treated with ^90^Y in the study required hospitalization for complications after treatment. A totally outpatient regimen was possible in 78% of the treated patients. This outpatient approach is in contrast to the prior use of ^131^I tositumomab at high administered radioactivity doses or ^131^I MIBG, where hospitalizations for radiation isolation are required for days to weeks before patients can be discharged safely from the hospital [12,13].

The individualized dosimetry approach applied here allowed ^90^Y ibritumomab tiuxetan to be delivered safely with relatively modest toxicity, other than that expected to the bone marrow (which was reversed with stem cell transplant) in most cases. The maximum activity administered, 6.33 GBq (171 mCi), was greater than 5 times the FDA-approved maximum administered activity (32 mCi) for non-myeloablative treatment.

Notable is that the liver radiation-absorbed dose of 28 Gy in 4 patients was well tolerated. This single dose of internally administered radiation to the liver in patients without liver disease suggests the MTD lies above 28 Gy radio-antibody radiation delivery to the liver. We presume the liver uptake of the radio-antibody is uniformly delivered to the hepatocytes. These data build upon those reported by Cremonesi whose trial limited planned Y-90 zevalin dose delivery to the liver to 20 Gy [16]. Our data also build upon the results of Chiesa, whose highest radiation-absorbed dose to liver was 24.3 Gy, in a patient receiving 45 MBq/kg of Y90 zevalin [17]. Knowledge that the MTD to the liver from radio-antibodies labeled with Y90 exceeds 28 Gy expands our knowledge of liver radiosensitivity to internally administered radio-pharmaceuticals. Using conventional fractionated external beam irradiation, the maximum tolerated radiation-absorbed dose to the liver is estimated as 30 Gy [24]. Standard fractionation is typically delivered as 200 cGy/day × 5 days/week [25]. The initial dose rate from Y90 ibritumomab tiuxetan is likely somewhat higher than that of external beam irradiation due to the 64 h half-life of Y90, but the lack of hepatic toxicity with Y90 suggests that the internally delivered radiation is no more toxic than external beam radiation. While biologic effective dose (BED) is in theory more relevant, it is very hard to confidently determine BED due to uncertainties in underlying assumptions in the liver.

Dosimetry data to the liver from radiolabeled microspheres is difficult to directly compare to the more uniform delivery of radio-antibodies to the hepatocytes. Since microspheres are delivered intra-arterially and lodge in pre-capillary arterioles, they are expected to have a non-uniform dose delivery to the liver, with more dose to tumor than liver [20].

There have been multiple other studies in which ^90^Y ibritumomab tiuxetan has been combined at the standard dose of 14.8 MBq/kg with myeloablative chemotherapies and stem cell transplant [25,26,27]. These do not provide information about the MTD of ^90^Y ibritumomab tiuxetan monotherapy at myeloablative radiation dosing but have generally supported the safety of including standard dose ibritumomab tiuxetan RIT as part of a myeloablative treatment regimen in NHL.

^90^Y ibritumomab tiuxetan has been given at myeloablative doses as a single agent on an inpatient basis. Ferruci et. al. performed a dose-escalation study in 13 patients at the following dose levels, 29.6 MBq/kg (0.8 mci/kg), 44.4 MBq/kg (1.2 mCi/kg), and 55.5 MBq/kg (1.5 mCi/kg), expanded upon by Cremonesi [16,28]. Using the planar dosimetry approach, they also showed considerable variability in the predicted organ radiation-absorbed doses per unit activity administered. They also found that the greatest radiation-absorbed dose per unit activity was to the liver, with hepatic radiation-absorbed dose consistently exceeding that delivered to the lungs or the myocardium. While fairly large administered radio-activities of ^90^Y were administered, median liver radiation-absorbed doses in the three dose cohorts were 7.2, 11.7, and 13 Gy, although these doses were not prospectively determined. In one patient, an estimated liver dose of 23.4 to 31 Gy (depending on method) was reportedly delivered. Two patients developed febrile neutropenia, one patient had HSV pneumonia, one patient had bacterial pneumonia, and one patient had reactivation of hepatitis C with eventual death without evidence of residual tumor at death. The authors concluded that the highest administered activity dose was appropriate only for patients who had had 3 or fewer regimens of prior chemotherapy and whose platelet counts were >150,000 mm^3^. They suggested that the lower dose of 44.4 MBq/kg (1.2 mCi/kg) was appropriate for patients who were more heavily pre-treated or whose platelet counts were in the 100,000–150,000 range.

Given the considerable variability in organ dose reported by Ferruci et al., in two instances, one at the highest planned dose (55.5 MBq/kg) and in one case at the middle dose level (44.4 MBq/kg), the liver radiation-absorbed dose exceeded the upper limit they planned to give. For example, at the 55.5 MBq/kg range, the authors predicted liver radiation-absorbed doses of 7.9 to 31 Gy [27]. The variability in radiation-absorbed dose to organs per unit administered activity they observed is consistent with the up to 3.5-fold variance we saw in our study in the radiation-absorbed dose per unit activity to the liver/kg body weight using our hybrid SPECT/CT-planar method. We have previously shown that this hybrid dosimetry approach is more accurate than conventional planar methods [19,22], possibly accounting for some of the differences between the two reports in absolute radiation-absorbed dose delivery. Cremonesi has pointed out the considerable variability in radiation-absorbed dose estimation depending on the dosimetry approach chosen for planar imaging. These differences are less in larger organs, however [16].

The determination of an MTD based on administered activity per unit body weight is challenging if the radiation-absorbed dose delivered is highly variable as a function of activity per unit mass. This is clearly the case with ^90^Y ibritumomab tiuxetan. We targeted delivery of up to 30.5 Gy to the liver without hepatic toxicity using organ-based dosimetry. While only one patient was treated at this planned dose level, three were treated at the 28 Gy level and 6 at the 24 Gy level, and had no evidence of liver toxicity. Thus, the MTD of 13 Gy to the liver proposed by Ferruci et al. is less than half of the dose we were able to safely deliver in our study, and is well under the hepatic radiation-absorbed dose accepted as the MTD for external beam radiation [24]. While the administered activity/weight-based MTD that they propose may be sufficient to deal with the occasional patient with a high hepatic radiation-absorbed dose, it appears to substantially underestimate the quantity of radioactivity that could be delivered to the liver if accurate prospective dosimetry was performed to guide the administered activity.

Devizzi et al. conducted a study in which chemotherapy was first given to NHL patients, followed by administration of a 29.6 to 44.4 MBq/kg dose of ^90^Y ibritumomab tiuxetan, followed by stem cell transplant [29]. Toxicity was generally acceptable [29,30]. In this study, planar dosimetry was performed. The liver was the organ receiving the highest radiation-absorbed dose per unit activity (except for spleen), and in 4 patients treated at the 44.4 MBq/kg dose, a median liver radiation absorbed dose of 20.9 Gy with a range of 15.4 to 24.3 Gy was seen without hepatic toxicity. The authors predicted that they could have given higher doses to the liver based on calculations of relative biological efficacy [17,29]. Our data support their view. It is difficult to compare other toxicities to our study in that the patients studied by Devizzi had 6 cycles of chemotherapy before the RIT dose and a tandem stem cell transplant procedure, and ours had only rituximab therapy.

We did not escalate the dose sufficiently to determine the MTD in our study. Unfortunately, due to a change in ownership of the company making the drug ^90^Y ibritumomab tiuxetan, our study was terminated prematurely. Liver tolerance of these radiation-absorbed doses was quite acceptable, suggesting room for further escalation of dose to the liver before an MTD is achieved. Unlike the study by Ferruci, we had no challenges with engraftment of stem cell reinfusions and cannot confirm that a low starting platelet count is a contraindication to high-dose therapy if there are adequate numbers of stem cells infused. Our entry criteria allowed patients with platelet counts of >75,000 mm^3^ and higher to enter the study, as well as patients who had up to 35% bone marrow involvement.

^90^Y ibritumomab tiuxetan has been investigated as a stem cell transplant agent in patients with multiple myeloma in combination with high-dose melphalan. Using ^111^In ibritumomab tiuxetan for dosimetry and cohorts with radiation-absorbed doses to the liver of 10, 12, 14, 16, 18, and 20 Gy, a dose limit was identified in 3 patients, yielding a maximum tolerated liver radiation-absorbed dose of 18 Gy for this treatment [31].

Our observations on dosimetry support the MTD of ^90^Y ibritumomab given without immediately preceding chemotherapy as being greater than 28 Gy to the liver. Our study also showed considerable biological and dosimetric variability in the absorbed dose to the liver and other tissues as a function of the activity per unit mass of ibritumomab tiuxetan administered [29]. We believe our estimates to radiation-absorbed dose variability are more reliable than those previously reported with only planar imaging. These point to the clear advantage of patient-specific dosimetry to safely optimize dose escalation.

Winter et al. integrated a somewhat higher than standard dose ibritumomab tiuxetan therapy with chemotherapy as part of a myeloablative transplant regimen [32]. They used personalized dosimetry to integrate ibritumomab tiuxetan RIT into the high-dose BEAM chemotherapy regimen for patients with relapsed or refractory CD20+ non-Hodgkin’s lymphoma (NHL). Several dose cohorts were studied with up to 17 Gy to critical organs allowed in the highest dose cohort. The therapeutic dose of ^90^Y ibritumomab tiuxetan was followed by high-dose BEAM and autologous transplantation. The authors found two dose-limiting toxicities at the 17 Gy dose level, which made 15 Gy the recommended maximum tolerated radiation-absorbed dose. These toxicities were a death at day 10 of pneumonia with renal failure, while another patient at the 17 Gy dose developed septic pulmonary emboli. The authors found that a weight-based strategy using 29.6 MBq/kg (0.8 mCi/kg) would have resulted in a wide range of radiation-absorbed doses to the liver and other tissues: nearly 25% of patient cases would have received 17 Gy or more, and many would have received less than 10 Gy. They concluded that dosimetry is required to avoid toxicity and undertreatment. One would expect the MTD of ibritumomab tiuxetan to be lower when combined with chemotherapy than when given as a monotherapy. The toxicities they observed were consistent with neutropenia, but were not hepatic in nature. Given the small size of our study and the heterogeneous makeup of our patients, it is difficult to make too many conclusions about response rate. However, our 88% response rate with 13 CR and 3 PR out of the 18 patients receiving the therapeutic dose of ^90^Y ibritumomab tiuxetan compares favorably with the 61.5% response rate (6 CR, 2 PR) in the 13 patients treated with high-dose ibritumomab tiuxetan monotherapy by Ferruci et al. [28]. Our response rate also compares favorably with the somewhat lower response rates reported for the lower, standard dose (11.1 to 14.8 MBq/kg) ibritumomab tiuxetan therapy of 40% to 60% [33]. We did not observe a clear difference in response or duration, based on the different dose cohorts studied, so are unable to conclude if higher radiation-absorbed doses truly resulted in superior anti-tumor activity. Concerning, however, was the relatively short duration of many of the responses. This duration of response may be shorter than that which has been reported by Gopal, Press, and colleagues using ^131^I tositumomab high-dose therapy of lymphoma [12,13]. With a median follow-up time of 42 months, the estimated overall and progression-free survival rates were 68% and 42% respectively, which are possibly longer than those seen in our group. It is possible that the differing beta particle energies between ^131^I and ^90^Y could be contributing to this possible difference. It is also possible that our patients’ substantial rituximab treatments before RIT reduced tumor targeting of the ^90^Y ibritumomab tiuxetan.

One patient in our study developed a myelodysplastic syndrome. This is a serious toxicity outcome and is well-known to follow myeloablative transplants, regardless of the method of myeloablation. Our observation is consistent with that of Guidetti et al., who evaluated the hematopoietic damage and incidence of secondary myelodysplastic syndrome and acute myelogenous leukemia (sMDS/AML) in patients who received myeloablative doses of the radiolabeled antibody ^90^Y ibritumomab tiuxetan. The occurrence of sMDS/AML was investigated prospectively in 53 elderly patients with non-Hodgkin’s lymphoma (NHL) who underwent an autograft after high-dose RIT (HD-RIT) myeloablative conditioning with Y-ibritumomab tiuxetan. The 5-year cumulative incidence of sMDS/AML was 8.29% [30].

It is important to point out that our approach to ibritumomab tiuxetan therapy differed from standard approaches in several ways: (1) the absolute radiation-absorbed dose given was much higher, and (2) the patients received weekly injections of 375 mg/m^2^ rituximab for 4 weeks preceding the radiolabeled antibody doses. The purpose of these infusions in patients who were still judged to be rituximab-sensitive was to purge tumor cells from the circulation before harvesting the stem cells for transplant. While this is a logical approach to clear circulating tumor cells, the amount of unlabeled rituximab given in the 4 weeks preceding the RIT was substantially higher than that typically given prior to RIT, which is 250 mg/m^2^ × 2. We did not infuse rituximab immediately prior to either of the ibritumomab tiuxetan infusions as rituximab would remain present in the circulation at the time of the RIT doses based on the typical half-life of rituximab [34]. We did not measure rituximab levels at the time of radio-antibody infusion. It is known that very high levels of rituximab may reduce the targeting of radiolabeled anti-CD20 antibodies to tumor in animal models [35]. In addition, there is some suggestive data that rituximab may have some paradoxical radioprotective effects, at least in vitro, which might reduce the biological efficacy of the radiation delivery to tumor [36]. Other considerations include the possibility that the range of the high-energy beta particle of ^90^Y may be too long to efficiently deposit energy into small tumor foci. These small foci might be better treated with a less energetic beta particle such as that from ^131^I or alternative radiotracers with lower beta energies [37]. A potential limitation of our study is that our predicted radiation dose to the liver is estimated from the ibritumomab tiuxetan imaging studies and not from direct measurement of Y90 activity in the liver, though in general, ^111^In antibodies are reasonably accurate predictors of ^90^Y antibody pharmacokinetics [38]. Since this was not a phase II study nor did we reach the MTD of ^90^Y ibritumomab tiuxetan, it was not possible to make definitive conclusions on the therapeutic efficacy of high-dose ^90^Y Ibritumomab tiuxetan in humans, as we did not treat a patient cohort at the MTD, though the treatment was clearly quite active.

## 5. Conclusions

Precision, prospective, SPECT-CT + planar organ dosimetry-based outpatient myeloablative radioimmunotherapy, with autologous stem cell transplantation (ASCT) with high-dose ^90^Y-RIT, was technically feasible with outpatient ASCT support in 18 patients with chemotherapy refractory or resistant CD20-positive non-Hodgkin’s lymphoma in a two-center radiation-absorbed dose-escalation study. There was patient-to-patient heterogeneity in dosing to the liver of over 3.5-fold. This dosimetry-based approach allowed a substantial increase in administered activity and radiation-absorbed dose as compared to standard FDA-approved non-myeloablative dosing with manageable toxicity. Tumor response rates of 88% were seen, with most responses complete, though responses were not particularly durable. The MTD was not reached in this study despite delivery of a targeted 30.5 Gy to the liver as the drug became unavailable. Additional dose escalation, informed by patient-specific radiation dosimetry, to determine second organ toxicities in the setting of stem cell transplant support are in order to determine the maximum anti-tumor effect of this form of radioimmunotherapy in B cell lymphomas given at the MTD, with such data broadly informing other treatment studies with radiopharmaceuticals.

## 6. Key Points

Question: Can outpatient precision prospective SPECT/CT + planar organ dosimetry of In-111 ibritumomab tiuxetan be performed and allow safe dose escalation of ^90^Y Ibritumomab tiuxetan, at myeloablative doses, in non-Hodgkin lymphoma patients who then receive outpatient stem cell transplants? What is MTD to liver, and is there therapeutic activity?

## Figures and Tables

**Table 1:** Patient population and key demographic considerations. # = number.

**Gender**		# (%)
	Male	14 (58)
	Female	10 (42)
**Age**		years
	Median	55
	Range	44–70
**Diagnosis**		# (%)
	SLL	2 (8)
	Follicular Lymphoma	11 (46)
	Transformed or Mixed Follicular Lymphoma	5 (21)
	Mantle Cell Lymphoma	5 (21)
	Large B-Cell Lymphoma	1 (4)
**Prior Therapies**		# (%)
	Median	3
	Range	1–5

**Table 2:** Injected radioactivity quantities and targeted major organ radiation doses from these activities. # = patient number.

Patient #	Gender	Mass (kg)	Height (cm)	Prescribed AA (GBq)	Actual AA (GBq)	Target Liver AD (cGy)	Liver AD (cGy)	Heart AD (cGy)	Lungs AD (cGy)	Kidneys AD (cGy)	Spleen AD (cGy)	MBq/kg for 10 Gy Liver Dose
1	M	92.3	175		1.42	-	1269	261	291	301	388	12.1
2	F	59	165		0.88	-	402	276	247	249	408	37.1
3	M	101.3	175		1.52	-	363	74	167	171	2647	41.4
4	M	76.5	179	3.36	3.35	1800	2112	1061	1155	1087	1173	20.8
5	F	59	150	2.52	2.46	1800	1180	610	563	764	902	35.4
6	M	77.6	176	3.11	3.08	1800	2053	613	652	1132	1054	19.3
7	M	63	170	4.53	4.50	2400	2500	1440	1898	1322	1894	28.6
8	F	71.1	159	2.81	2.80	2400	1348	642	621	677	685	29.3
9	F	69	145	2.58	2.59	2400	2112	787	734	594	1109	17.8
10	M	82.5	178	3.87	3.49	1800	1672	897	1020	817	1150	25.3
11	F	68.5	160	3.01	2.98	1800	1378	2431	918	694	1073	31.6
12	F	86.7	160	3.58	3.55	2400	1678	1123	1211	1133	2337	24.4
13	F	70.2	157	3.12	3.19	2400	1380	879	950	898	1177	32.9
14	M	68.8	177.8	4.26	4.24	2400	2261	1410	1327	1235	2249	27.3
15	F	95.4	160	5.10	5.04	2800	2250	1072	1318	1129	2017	23.5
16	M	85	-	5.21	5.29	2800	2848	1411	1911	845	1147	21.9
17	M	90.7	184.2	6.32	6.33	2800	2806	1635	2196	948	1520	24.9
18	M	81	173	6.98	6.26	3050	2734	1945	1946	1202	2030	28.3
mean		77.6	167.3				1796.8	1031.4	1062.5	844.3	1386.7	26.8
median		77.1	170.0				1865.4	978.8	984.8	871.6	1161.9	26.3
SD		12.5	11.2				744.4	609.2	616.9	347.4	663.8	7.3
min		59.0	145.0				363.3	73.9	166.5	170.7	388.4	12.1
max		101.3	184.2				2847.7	2430.6	2196.4	1321.8	2647.2	41.4

**Table 3:** Response rates and duration by dose cohort.

Cohort	Number of Patients	Median (Months)	Range (Months)
14.18 Mbq/Kg	3	12.6	6.1–18.4
14–19 Gy	5	9.0	0.1–9.7
24 Gy	6	9.3	0.9–40.2
28 Gy	3	23.0	9.0–>37
30.5 Gy	1	>27	>27
Overall	18	>13	0.1–40.2

## Data Availability

The data presented in this study are available on request from the corresponding author.
